# Metabolic Profiles of Brain Metastases

**DOI:** 10.3390/ijms14012104

**Published:** 2013-01-22

**Authors:** Torill E. Sjøbakk, Riyas Vettukattil, Michel Gulati, Sasha Gulati, Steinar Lundgren, Ingrid S. Gribbestad, Sverre H. Torp, Tone F. Bathen

**Affiliations:** 1 MI Lab, Department of Circulation and Medical Imaging, Norwegian University of Science and Technology (NTNU), Trondheim N-7491, Norway; E-Mails: muhammad.r.vettukattil@ntnu.no (R.V.); michelg@stud.ntnu.no (M.G.); ingrid.s.gribbestad@ntnu.no (I.S.G.); tone.f.bathen@ntnu.no (T.F.B.); 2 St. Olavs University Hospital HF, Trondheim N-7006, Norway; 3 Department of Neuroscience, NTNU, Trondheim N-7491, Norway; E-Mail: sashagulati@hotmail.com; 4 Department of Neurosurgery, St. Olavs University Hospital HF, Trondheim N-7006, Norway; 5 Department of Oncology, St. Olavs University Hospital HF, Trondheim N-7006, Norway; E-Mail: steinar.lundgren@stolav.no; 6 Department of Cancer Research and Molecular Medicine, NTNU, Trondheim N-7491, Norway; 7 Department of Pathology and Medical Genetics, St. Olavs University Hospital HF, Trondheim N-7491, Norway; E-Mail: sverre.torp@ntnu.no; 8 Department of Laboratory Medicine, Children and Women’s Health, NTNU, Trondheim N-7491, Norway

**Keywords:** magnetic resonance, spectroscopy, high-resolution, metabolite, *ex vivo*, cancer, carcinomas, metastasis

## Abstract

Metastasis to the brain is a feared complication of systemic cancer, associated with significant morbidity and poor prognosis. A better understanding of the tumor metabolism might help us meet the challenges in controlling brain metastases. The study aims to characterize the metabolic profile of brain metastases of different origin using high resolution magic angle spinning (HR-MAS) magnetic resonance spectroscopy (MRS) to correlate the metabolic profiles to clinical and pathological information. Biopsy samples of human brain metastases (*n* = 49) were investigated. A significant correlation between lipid signals and necrosis in brain metastases was observed (*p* < 0.01), irrespective of their primary origin. The principal component analysis (PCA) showed that brain metastases from malignant melanomas cluster together, while lung carcinomas were metabolically heterogeneous and overlap with other subtypes. Metastatic melanomas have higher amounts of glycerophosphocholine than other brain metastases. A significant correlation between microscopically visible lipid droplets estimated by Nile Red staining and MR visible lipid signals was observed in metastatic lung carcinomas (*p* = 0.01), indicating that the proton MR visible lipid signals arise from cytoplasmic lipid droplets. MRS-based metabolomic profiling is a useful tool for exploring the metabolic profiles of metastatic brain tumors.

## 1. Introduction

Metastases are the most common cancers of the central nervous system and a feared complication of systemic cancer with significant morbidity and a poor prognosis. The true incidence is unknown and probably underestimated [1,2]. Brain metastases from lung, melanoma, breast, kidney and colon carcinomas are most frequently observed [1]. Treatment modalities include chemotherapy, stereotactic radiosurgery, whole brain radiotherapy and surgery. The treatment offered depends on the origin of the metastases, clinical condition of the patient, tumor size and also number, depth and mass effect of lesions [3,4]. Age, Karnofsky performance status (KPS), number of intracranial metastases and the presence of extracranial metastases are established prognostic factors [5,6]. A recent study of patients with brain metastases treated with radiotherapy showed that more than 30% died within three months [7]. Studies of a similar patient cohort, stratified by graded prognostic assessment and treated by open surgery, showed a three-months’ mortality rate of 17% [8,9]. Hence, there is a substantial need for improved methods to identify prognostic markers that can help to risk-stratify these patients.

Magnetic resonance (MR) metabolomics of intact tissue is being increasingly recognized as a promising tool within oncology. High resolution magic angle spinning (HR-MAS) magnetic resonance spectroscopy (MRS) is a novel analytical method that provides detailed biochemical information of intact tissue samples. Even though experimental conditions might cause minimal structural damage, post HR-MAS analyses enable reasonable comparisons of metabolic information with histopathological data [10] or molecular methods, such as gene expression [11]. Several studies suggest that metabolic profiling provides valuable information in cancer diagnostics [12,13], characterization and treatment monitoring [14,15]. Brain metastases of different origin have previously been described by HR-MAS to consist of more than 30 metabolites, dominated by lipid signals in many of the investigated samples [16]. The metabolic profile was found to reflect the origin of the metastases and to correlate to patient prognosis [16]. Metabolic profiling by HR-MAS MRS might contribute to improve our understanding of the biology of the different brain metastasis.

The objectives of this study were to characterize the metabolic profile of brain metastases of different origin using HR-MAS MRS, to correlate the metabolic information to clinical and histopathological parameters and to further explore the source of MR visible lipids.

## 2. Results and Discussion

### 2.1. Histopathology

The routine histopathology assigned the samples of brain metastases to originate from malignant melanomas (*n* = 6), various carcinomas (breast (*n* = 6), colorectal (*n* = 12), lung (*n* = 20), uterine cervical (*n* = 2), esophageal (*n* = 1), renal (*n* = 1)) and one of unknown origin (*n* = 1). In addition to the one of unknown origin, the metastases originating from uterine cervical, esophageal and renal carcinomas were excluded from the study due to the low number of samples (*n* < 3). The characteristics of the remaining patient cohort (*n* = 44) and samples are presented in Table 1.

Post HR-MAS histological assessment was performed in all samples (*n* = 44) and concurred with the initial histopathological diagnoses. Five samples, where their post HR-MAS histology predominantly composed of brain tissue or fibrotic tissue, were excluded from further statistical analyses. The relative content of tumor tissue, necrosis and fibrosis in the remaining cohort (*n* = 39) is shown in Table 2. The non-destructive nature of HR-MAS spectroscopy of tissue allowed precise characterization of the same tissue samples by histopathological post-processing, and further investigations, such as gene expression studies, can also be performed [12,17].

### 2.2. MR Metabolomics

**Figure 1:**
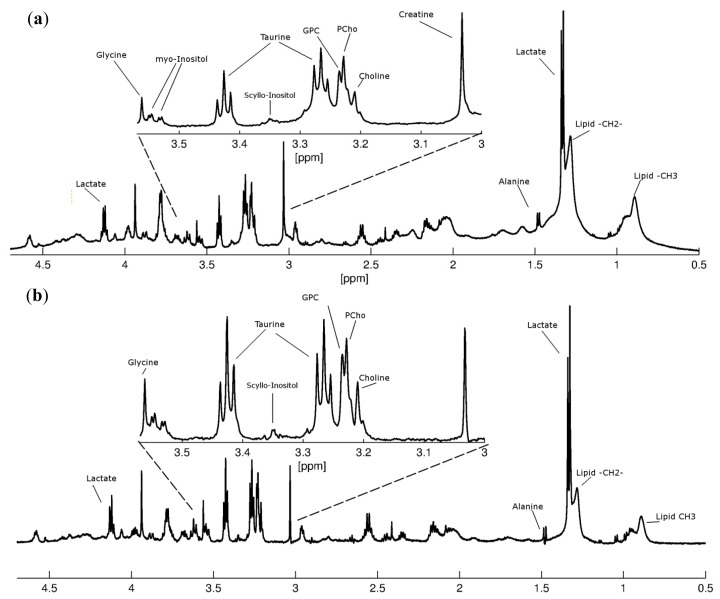
Representative proton HR-MAS spectra from a patient with brain metastasis from lung carcinoma obtained as (**a**) pre-saturated, single-pulse spectrum and (**b**) spin-echo spectrum, showing assignment of various metabolites. The enlarged ppm region 3.6–3.0 ppm shows more details of glycine (Gly), myo-inositol (myo-In), taurine (Tau), scyllo-inositol (scy-In), glycerophosphocholine (GPC), phosphocholine (PCho), choline (Cho) and creatine (Cr).

The type of information obtained from HR-MAS strongly depends on the type of MR experiment. In this study, pre-saturated, single-pulse (zgpr) experiments were performed to obtain water suppressed spectra showing various metabolites (Figure 1a). To remove the broad signals from lipids and macromolecules, subsequent spin-echo (cpmgpr) experiments were performed (Figure 1b). Separate multivariate analyses were done on the spectral data obtained from both of these experiments.

#### 2.2.1. Brain Metastases and Lipid Metabolites

In this study, we observed high lipid levels in the spectra from many of the brain metastasis samples. MR-visible lipids are triglycerides and cholesterol esters that accumulate in lipid droplets (LD) arising in response to unfavorable conditions in the tumor microenvironment [18–21]. *In vivo* and *ex vivo* MRS studies of brain tumors have demonstrated that gliomas and brain metastases are characterized by dominating lipid signals [21–23]. A correlation between these lipid signals and amount of necrosis has been demonstrated in *ex vivo* studies of gliomas [24–26]. High levels of MR-visible lipids in metastases may therefore also be related to the amount of necrosis [27]. However, cellular response to stressful stimuli from the tumor microenvironment can result in accumulation of neutral lipids in cytoplasm. It has been shown that MR-visible lipid signals in non-necrotic brain tumor biopsies are mobile lipid droplets of cytoplasmic origin [21,25]. Further, apoptosis and hypoxic cells have been reported to contribute to elevated spectral lipid signals [28].

**Figure 2:**
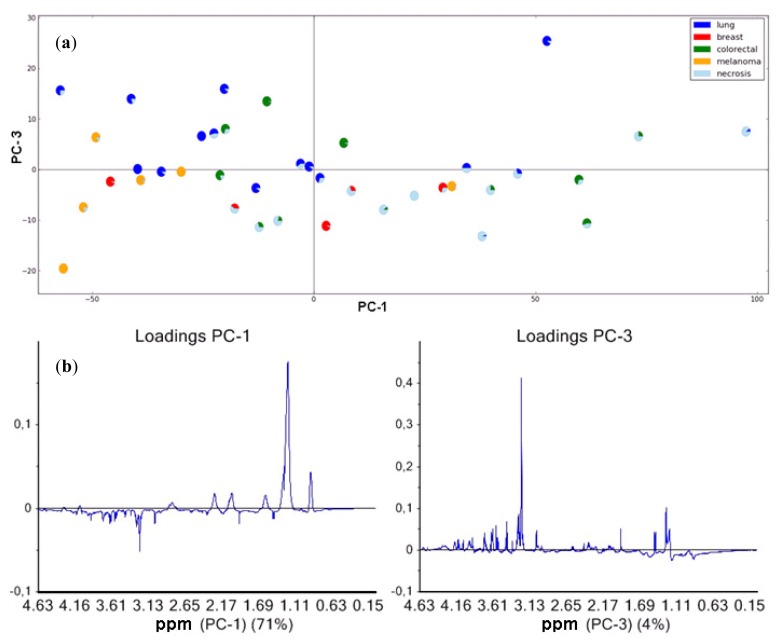
(**a**) Principal component analysis (PCA) score plot of PC-1 (explained variance 71%) and PC-3 (explained variance 4%) based on single-pulse spectra (*n* = 39) showing the distribution of the metastases from four different primary cancer diagnoses; lung (blue), breast (red), colorectal (green) and melanoma carcinomas (yellow). The light blue colored part of the circles reflects the content of necrotic tissue determined by HES staining post HR-MAS; (**b**) Loading plots for PC-1 and PC-3; Samples with high score values for PC-1 have higher lipid levels. High score values for PC-3 are mainly due to high levels of phosphocholine (PCho).

In our study, we found a significant correlation (*p* < 0.01, Spearman two-tailed) between spectral lipid signals and the percentage of necrotic tissue in the brain metastases. To explore the variation in the spectral data, principal component analyses (PCA) were performed. Figure 2a shows the PCA score plot of the first (PC-1) *versus* the third (PC-3) principle component from the analyses of the single-pulse spectra. The necrotic fraction in the samples increases along the PC-1 axis of the score plot. The corresponding loading profile (Figure 2b) shows that high lipid levels are associated with necrosis. Partial least square (PLS) regression of the same spectra showed a correlation between the metabolic profile and necrotic fraction (*r*^2^ = 0.31), proved significant by permutation testing (*n* = 10,000, *p* = 0.047). This is in accordance with several previous studies of brain tumors, where MR-visible lipid signals correlated with necrosis [22,25,27,29,30]. The sample scattering along the PC-3 direction in Figure 2a is mainly due to differences in phosphocholine (PCho). Clustering according to the origin of the metastases is not so distinct; however, the brain metastases from malignant melanoma tend to cluster as a group of low lipid containing samples with low values of both PC-1 and PC-3 (Figure 2). The metastases from breast carcinomas appear with low PC-3, but both low and high PC-1, while both metastases from lung and colorectal carcinomas appear to be very heterogeneous as they disperse over the scatter plot. A recent HR-MAS study showed that histological subtypes of patients with primary lung carcinomas can be identified by PLS-analyses of MR spectra [31]. Most of the samples of brain metastases from lung carcinomas in our study were adenocarcinomas and, hence, the cohort is too small for a meaningful subtype analysis. The PCA demonstrate the large impact of lipids on the distribution of the samples. However, metastatic tumors with a higher degree of estimated necrosis by histopathology cluster together irrespective of their primary origin.

Differences in lengths and numbers of double bonds in fatty acyl chains generate MR signals at different chemical shifts (ppm). The broad signal at 0.9 ppm is the joint signal for the terminal methyl group (–CH_3_), found in all fatty acyl chains. When the spectral lipids at the ppm values 1.3, 1.55, 2.05, 2.25 and 2.82 were scaled relative to the signal at 0.9 ppm and compared to the necrotic fraction in the samples, only the 1.3 ppm signal correlated significantly with necrosis. Since the signal at 1.3 ppm ((–CH_2_)*_n_*) indicates the degree of saturation in fatty acids, this indicates an abundance of saturated fatty acid in necrosis.

To further explore and compare any prognostic value of the metabolic information, the histological and clinical parameters (tumor tissue fractions, age and survival) were related to the metabolic profiles by PLS. No significant correlations between metabolic profiles and patient age or survival at three or five months after surgery was observed, and previous intriguing results were thus not confirmed [16]. This is probably due to the large heterogeneity of this patient cohort regarding age, performance score, primary cancer and numbers of brain metastases. Compared to the previous study, we have a higher number of patients, indicating the need for further verification in future studies in larger cohorts.

#### 2.2.2. Nile Red Analysis of Lipid Droplets

**Figure 3:**
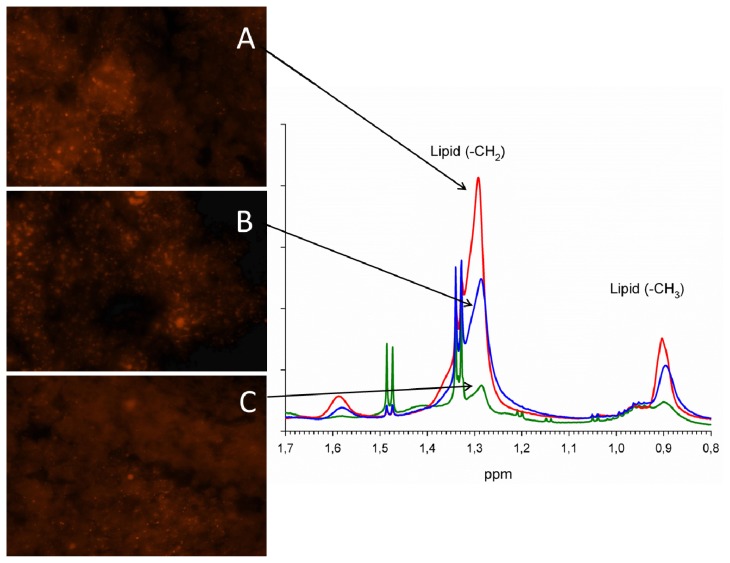
Nile Red fluorescent-stained images showing lipid droplets in three brain metastases originating from lung carcinoma (A, B, C) and their corresponding mean normalized single-pulse spectra (1.6–0.8 ppm), which demonstrate signal differences due to methylene (1.3 ppm) and methyl groups (0.9 ppm) in the mobile fatty acyl chains.

**Figure 4:**
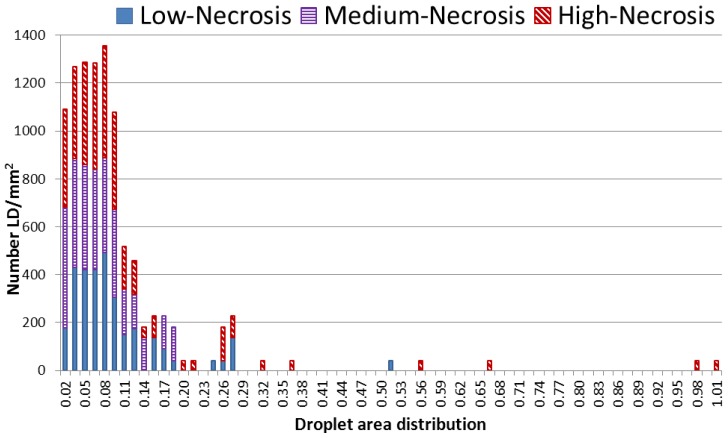
Distribution of lipid droplets for droplet area between 0 and 1.005 μm^2^ in bins of 0.015 μm^2^. The *x*-axis represents droplet area distribution and the *y*-axis—droplet density (number LD/mm^2^). The red, purple and blue bars represent the mean number of LD in high (>70%) medium (40%–70%) and low (<40%) necrosis, respectively.

MR visible lipids in brain metastases were further investigated by Nile Red staining to explore the origin of the lipid signals seen in the MR spectra. Nile Red is a selective stain for cytoplasmic lipid droplets and helps to study lipid droplet formation in pathological states [32]. The metastatic lung carcinomas samples were used as representative samples for this experiment due to their heterogeneous nature as observed in the PCA score plot with a wide variation in lipid levels (Figure 2). Analysis of the fluorescent microscopy images from Nile Red stained samples showed a varying amount and size of lipid droplets (LD). Figure 3 demonstrates Nile Red fluorescent-stained images of LD in sections of three samples with different metabolic patterns. LD were observed in focal necrotic parts of the sample sections, whereas tumor cells, stroma and brain cells were devoid of lipid droplets. A significant correlation between microscopically visible lipid droplets estimated by Nile Red staining and MR visible lipid signals (1.3 ppm) was observed in the metastatic lung carcinomas (*p* = 0.01). This is not previously shown for brain metastases, but is in consistence with previous findings in brain tumors [25,29]. Distributions of lipid droplet size and number are shown in Figure 4. Smaller lipid droplets are distributed equally among samples with low, medium and high necrosis, while larger lipid droplets only are present in samples with high necrotic fraction.

#### 2.2.3. Brain Metastases and Low-Molecular-Weight Metabolites

The PCA score plot of the single-pulse spectra (Figure 2) shows mainly an association between lipid levels and necrotic tissue, but also an inverse association to the level of PCho, lactate (Lac) taurine (Tau), glycerophosphocholine (GPC) and creatine (Cr) among brain metastasis of different origin. Tumors with a lower fraction of necrosis (Figure 2b), irrespective of metastatic origin, showed relatively higher levels of small molecular metabolites and lower levels of Lac and lipids, probably due to the dilution of metabolites in necrotic tumors [33].

**Figure 5:**
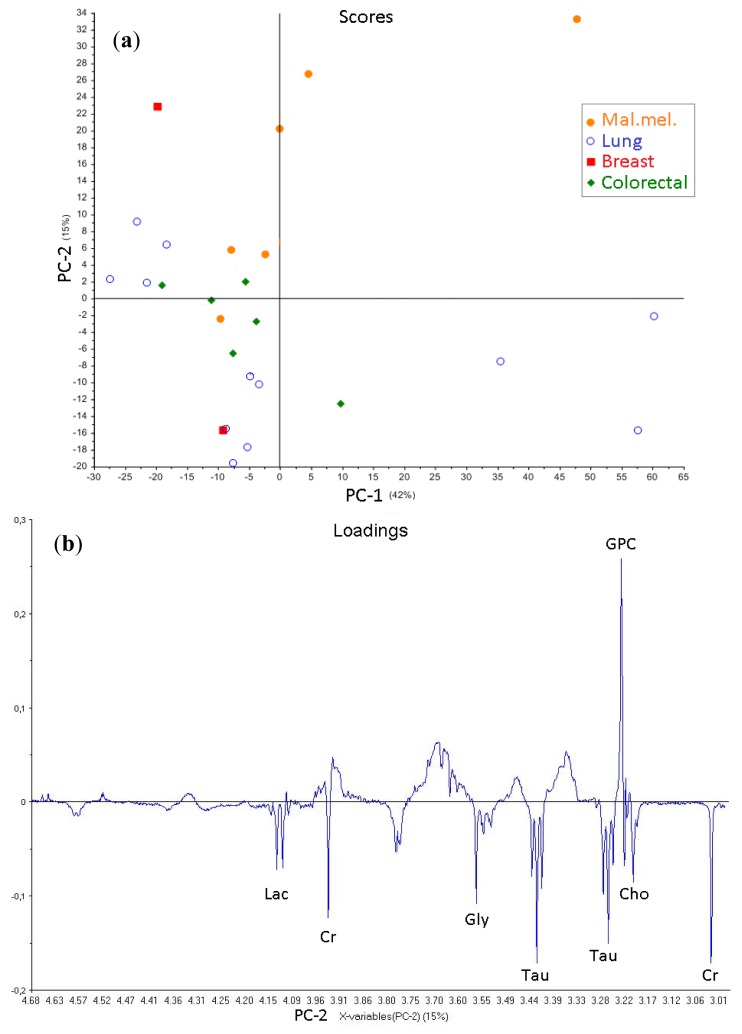
(**a**) PCA score plot showing PC-1 (42%) and PC-2 (15%) of cpmgpr spectra (ppm region 4.68–2.98) from brain metastases with less than 50% necrotic tissue (*n* = 26); (**b**) The loading plot of PC-2 indicates the metabolites GPC, Cr, Tau, Gly, Cho and Lac as important signals that divide the subtypes. The positive dominating signal in the loading plot for PC-1 (not shown) was PCho, explaining the dispersion in PC-1 direction in the score plot.

In order to exclude the contributions from necrosis related metabolites and to evaluate the small molecular metabolites in detail, samples with low necrosis (<50%) were further explored using the macromolecule suppressed spin-echo spectra. PCA was performed using the region between 4.68 and 2.98 ppm to exclude remnant lipid signals. The resulting score plot of PC-1 and PC-2 (Figure 5a) and the corresponding loading plot (Figure 5b) indicated a higher amount of GPC in malignant melanoma metastases than those from colorectal and lung carcinomas. Elevated GPC levels may reflect alteration in the enzymatic activities that govern choline synthesis, degradation, phosphorylation and transport. Metastases from colon and lung carcinomas have similar metabolic signatures, and they cluster together in the lower half of the PCA score plot (Figure 5a), with relatively higher levels of Cr, Gly, Lac and Tau compared to melanomas. Metastases from lung carcinomas are more heterogeneous compared to those from colon carcinomas and have wide variations in PCho content and are distributed along PC-1 (Figure 5a). Previous HR-MAS studies comparing primary lung carcinomas and normal lung tissue showed an increased amount of Lac, PCho and GPC in the former, while primary colon carcinomas showed a higher content of Tau, PCho, Gly and Lac compared to normal colon tissue [10,34]. Some of these metabolites, which distinguish primary lung and colorectal cancers from healthy tissues, overlap with the metabolites we identified in our analysis. To gain knowledge about the role of these metabolites in cancer dissemination, comparative metabolomic studies, including both primary and metastatic tissue, are necessary.

## 3. Experimental Section

The study protocol was approved by the Norwegian Social Science Data Services and the Regional Committee for Medical and Health Research Ethics. Written informed consent was obtained from all patients included.

### 3.1. Patient and Sample Characterization

Forty-nine patients with brain metastases (mean age 63, range: 38–81 years) scheduled for surgical resection of their metastases were enrolled to the study. Prior to surgery, the gross functional status of the patient was evaluated using KPS. All operations were performed under general anesthesia using the Sonowand neuronavigation system [35]. During surgery, most of the resected tissue was sent for routine histological analysis, while a small part of the fresh tissue was within two minutes (median time) snap-frozen in liquid nitrogen at −196 °C and stored in cryogenic vials. To prevent biochemical and structural degradation, the biopsy specimens were stored in liquid nitrogen until analysis by HR-MAS MRS. More details on the patients’ characteristics are shown in Table 1.

### 3.2. Sample Preparation and HR-MAS Experiments

The sample preparation was performed on an ice block to avoid degradation. Each sample was cut to fit a leak-proof insert (30 μL, Bruker: Kel-F) used in a zirconium HR-MAS rotor (4 mm). The insert was filled with 3 μL cold (about 4 °C) phosphate buffered saline based on deuterium oxide containing 4.5 mM TSP (trimethylsilyl-tetradeuteropropionic acid) and 20 mM sodium formate (CHNaO_2_). Mean sample weight was 9.1 ± 4.8 mg.

HR-MAS spectra were recorded at 4 °C using a 600 MHz spectrometer with a ^1^H/^13^C HR-MAS probe (Bruker Avance DRX600, Germany) spinning the samples at a rate of 5 kHz. A single-pulse sequence (Bruker: zgpr) and a water and lipid suppressing spin-echo Carr-Purcell-Meiboom-Gill sequence (Bruker: cpmgpr) were acquired and processed, as described elsewhere [16]. The spectral region between 4.6 and 0.5 ppm was used for chemometric analyses. Spectral assignments were performed based on previous HR-MAS MRS studies of brain tumors and metastases [16,36–38].

### 3.3. Histopathological Examination

The surgical specimens of the brain metastases were embedded in paraffin wax and cut in several sections (5 μm thick) before staining with hematoxylin, eosin and saffron (HES-staining) for the routine histological analysis. After HR-MAS analyses, the samples were immediately transferred to formalin for subsequent histopathological examination and were processed similarly. In the HES sections, the fraction of tumor tissue, necrosis, fibrosis and gliosis were assessed by a neuropathologist (SHT) (Table 1). The samples were divided in to three groups, high (>70%) medium (40%–70%) and low (<40%) necrosis, based on their necrotic content.

Nile Red staining was performed on a sub-cohort to further investigate the origin of the lipid signals seen in the MR spectra of brain metastases from lung carcinomas, and these samples were snap-frozen after HR MAS before being analyzed by Nile Red staining [32]. The metastatic lung carcinomas samples were used as representative samples for this experiment due to their expression in the PCA score plot. A fluorescence microscope (Nikon eclipse 90i) with an Exciter S 572filter and a camera were used to observe and photograph the Nile Red stained lipid droplets, as they appeared red luminous in the tissue. The field of view was kept at the region where the maximum numbers of lipid droplets where observed in the sample under an objective of 60× magnification. The processing and analysis of the Nile Red fluorescence images were performed using ImageJ software (Version 1.42q, National Institutes of Health, Bethesda, MD). After converting color images to 8-bit gray scale, the background fluorescence was removed with the provided ‘Subtract background’ (rolling ball, radius = 50 pixels) option in Image J, and the lipid droplets were segmented by applying a manual threshold. The number and area of lipid droplets were calculated by “Analyze Particles”.

### 3.4. MR Metabolomics

All spectra were peak aligned by the warping algorithm icoshift [39] in MatLab (version 7.9.0; The Math Works, Natick, MA, USA) and baseline offset was corrected. The spectral regions between 4.60–0.05 ppm and 4.68–0.05 ppm from pulse-acquired and spin echo spectra, respectively, were used as input for multivariate analyses. The spectral regions 3.69–3.62 ppm and 1.22–1.13 ppm were removed due to ethanol contamination. In order to eliminate the effect of differences in sample weights, the selected regions of the spectra were normalized by dividing each row of the data matrix by its average (mean normalization in Unscrambler (CAMO, version 10, Norway)).

Principal component analyses (PCA) of mean-centered data were performed to explore the variation in the spectral data. PCA reduces the dimensionality of the data and summarizes the similarities and differences between multiple MR spectra using score plots. New variables, which are linear combinations of the original spectra carrying the greatest variance within the data called principle components (PCs), are created. The first PCs will be in the direction of maximum variance in the data set, and samples with similar metabolic profiles will appear close to each other in the score plots of PCs. The variables or metabolites, which are responsible for the grouping of samples in the score plots, are identified by the corresponding loading plots of the PCs.

Partial least square discriminant analysis (PLS-DA) with cross validation was performed to investigate the relationship between the metabolic profiles (*x*) and clinical parameters (*y*). PLS models use both the *x*- and *y*-matrices simultaneously to find the latent variables (LVs) in *x* that best predict the *y*. PLS-DA attempts to discriminate between distinct classes and explains maximum separation between defined class samples in the data (*x*) [40]. To achieve a representative test and training set for the PLS-DA modeling, the sample selection algorithm Duplex was used [41]. The obtained test samples (*n* = 9) were kept out, while the training (*n* = 30) set was used to build the model. Both PCA and PLS-DA were performed using Unscrambler (CAMO, version 10, Norway). In order to evaluate whether sample classification was significantly better than random classification in two arbitrary groups, a permutation test was performed with 1000 permutations [42].

To study associations between lipid signals, PCA score values and necrosis, statistical analyses were done using IBM SPSS Statistics 19 (New York, NY, USA). All statistical tests used were non-parametric, two-sided, where *p*-values below 0.05 were considered as statistical significant.

## 4. Conclusions

In this study, we explored the metabolic profiles of metastatic brain tumors of different origin. Malignant melanomas were metabolically distinct, with a higher amount of GPC, while other subtypes were metabolically more heterogeneous. A significant correlation between lipids and necrosis in the metastatic tumors were observed. The study also confirms the cytoplasmic origin of MR visible lipid signals in metastases. However, there were no significant correlations between metabolic profiles and patient age or survival. MR spectroscopy is a useful tool for exploring the metabolic profiles of metastatic brain tumors, and future comparative metabolomic studies involving both primary and metastatic tissue may shed light on the role of metabolites in cancer dissemination.

## Figures and Tables

**Table 1:** Patient and sample characteristics (*n* = 44).

	*Origin of Carcinomas*				
	
Variables	Total cohort	*Lung*	*Colorectal*	*Breast*	*Melanoma*
Numbers	44	20	12	6	6
Female/Male	20/24	9/11	5/7	6/0	0/6
Median Age at Surgery (Range)	63 (37–80)	69 (38–80)	63 (43–81)	53 (45–66)	65 (53–76)
KPS%:					
90–100	13	5	2	3	3
70–80	25	12	8	2	3
<70	6	3	2	1	0
Five Month Postoperative Survival	28	12	9	4	3

KPS = Karnofsky Performance Status (0–100).

**Table 2:** Origin and mean percentages (± SD) of tumor, necrotic and fibrotic tissue of the brain metastases samples (*n* = 39), described by HES-staining after high resolution magic angle spinning (HR-MAS) analysis.

Origin Carcinomas of the Brain Metastases:	Tumor (%)	Necrosis (%)	Fibrosis (%)
*Lung* (*n* = 17)	49 ± 31	36 ± 34	12 ± 17
*Colorectal* (*n* = 11)	35 ± 24	48 ± 32	14 ± 23
*Breast* (*n* = 5)	25 ± 5	34 ± 30	35 ± 27
*Malignant Melanoma* (*n* = 6)	83 ± 15	8 ± 8	10 ± 15

*n* = number, HES = hematoxylin, eosin and saffron staining.
